# Unraveling Cuticle Formation, Structure, and Properties by Using Tomato Genetic Diversity

**DOI:** 10.3389/fpls.2021.778131

**Published:** 2021-11-29

**Authors:** Johann Petit, Cécile Bres, Nicolas Reynoud, Marc Lahaye, Didier Marion, Bénédicte Bakan, Christophe Rothan

**Affiliations:** ^1^INRAE, Univ. Bordeaux, UMR BFP, Villenave d’Ornon, France; ^2^Unité Biopolymères, Interactions, Assemblages, INRAE, Nantes, France

**Keywords:** tomato, cuticle, natural diversity, mutant, fruit, cutin, structure, property

## Abstract

The tomato (*Solanum lycopersicum*) fruit has a thick, astomatous cuticle that has become a model for the study of cuticle formation, structure, and properties in plants. Tomato is also a major horticultural crop and a long-standing model for research in genetics, fruit development, and disease resistance. As a result, a wealth of genetic resources and genomic tools have been established, including collections of natural and artificially induced genetic diversity, introgression lines of genome fragments from wild relatives, high-quality genome sequences, phenotype and gene expression databases, and efficient methods for genetic transformation and editing of target genes. This mini-review reports the considerable progresses made in recent years in our understanding of cuticle by using and generating genetic diversity for cuticle-associated traits in tomato. These include the synthesis of the main cuticle components (cutin and waxes), their role in the structure and properties of the cuticle, their interaction with other cell wall polymers as well as the regulation of cuticle formation. It also addresses the opportunities offered by the untapped germplasm diversity available in tomato and the current strategies available to exploit them.

## Introduction

Cultivated tomato (*Solanum lycopersicum* L.) is a major horticultural crop that has long been a model for the *Solanaceae* crop species (tomato, potato, eggplant, pepper …) and for fleshy fruit development and disease resistance (Rothan et al., 2019). Tomato is very suitable for laboratory studies (grown in greenhouse, miniature cultivars, short life cycle, autogamy, and easy genetic transformation) has extensive genetic resources, high-quality reference genome (Tomato Genome Consortium, 2012), sequences of hundreds of accessions (Zhu et al., 2018; Gao et al., 2019; Alonge et al., 2020), and available phenotype and gene expression databases.^1^ Thanks to these resources and tools, genes underlying trait variations can be identified and their relationships with phenotypic variations can be established through *in planta* functional analysis.

In the last decades, the use of natural diversity available in tomato, such as the ripening mutants *ripening inhibitor* (*rin*) or *non-ripening* (*nor*), has been instrumental to decipher the regulation of ripening (Klee and Giovannoni, 2011). These advances have been aided by the development of tools for functional analysis of target genes including RNA interference (RNAi) and CRISPR/Cas9 gene editing systems. The molecular determinants of fruit skin formation, which is an essential protective barrier against pests, pathogens, and water loss, have begun to be explored more recently (Vogg et al., 2004; Lemaire-Chamley et al., 2005; Hovav et al., 2007; Mintz-Oron et al., 2008).

Not surprisingly, since the cuticle is a major component of the fruit skin and is associated with a wide diversity of major breeding targets including fruit appearance (color, glossiness, regularity…) and properties (shelf-life, fungal resistance, and cracking; Bargel and Neinhuis, 2005; Petit et al., 2017; Lara et al., 2019), it has received considerable attention in recent years. In this field of study, tomato holds a prominent position among fleshy fruits because of its thick, astomatous, and easy-to-peel cuticle (Petit et al., 2017). Additionally, the wide diversity in cuticle architecture and composition found in wild tomato relatives (Yeats et al., 2012a; Halinski et al., 2015; Fernandez-Moreno et al., 2017) can be exploited for the discovery of novel cuticle-associated genes (Hovav et al., 2007; Zhang et al., 2021). Tomato has therefore become a model for the study of cuticle formation in plants. In the recent years, molecular determinants of cuticle have been identified and genetically altered lines have been produced, enabling the exploration of cuticle structure, properties, interactions with other cell wall components, and relationships with epidermal patterning. Table 1 summarizes the major findings on known tomato cuticle-associated genes, the pathway, or biological process in which they are involved and the main alterations produced by their mutation or de-regulation. This mini-review focuses on the strategies, resources, and tools used to reveal their role, providing examples, and considers future goals and developments.

**Table 1:** Genes involved in cuticle formation and properties studied in tomato.^*^

Gene-locus^a^	Solyc^b^	Type^c^	Function	Species^d^	Cultivar-accession^e^	Genetic variation origin/Allele^f^	Cuticle-associated traits	References
*SlCYP86A69*L	08g081220	Enzyme	Cutin monomer biosynthesis	*Sl*L	MT	EMS/*cd3/cyp86a69*L	Fruit cuticle thickness and properties; cutin content; pathogen susceptibility	Isaacson et al., 2009; Shi et al., 2013; Buxdorf et al., 2014
*SlGPAT6*L	09g014350	Enzyme	Cutin monomer biosynthesis	*Sl*L	MT	EMS/*cud1/gpat6-a*L	Fruit cuticle thickness and properties; cutin content; epidermal patterning; cell wall properties; pathogen interaction	Petit et al., 2016; Philippe et al., 2016; Fawke et al., 2019; Moreira et al., 2020
*SlABCG36/42*L	05g018510 06g065670	Transporter	Cutin monomer transport	*Sl*L	MT	RNAi silencing	Fruit cuticle thickness; cutin content and composition	Elejalde-Palmett et al., 2021
*SlCUS1*L	11g006250	Enzyme	Cutin polymerization	*Sl*L	MT	EMS/*gdsl2b/cus1-a*L	Fruit cuticle thickness and properties; cutin content; epidermal patterning; susceptibility to pathogens	Petit et al., 2014; Moreira et al., 2020; Segado et al., 2020
				*Sl*L	M82	EMS/c*d1/cus1*L		Isaacson et al., 2009; Yeats et al., 2012b
				*Slc*L	WVa106	RNAi silencing		Girard et al., 2012; Philippe et al., 2020a
*SlDCR*L	03g025320	Enzyme	Cutin polymerization	*Sl*L	M82	RNAi silencing	Flower and leaf fusion; fruit cracking and suberin formation	Lashbrooke et al., 2016
*SlCER6/SlKCS6*L	02g085870	Enzyme	Wax biosynthesis	*Sl*L	MT	*Ds*-insertion/*cer6*L	Flower fusion; fruit dehydration; alkanes and terpenoids	Vogg et al., 2004; Leide et al., 2007; Smirnova et al., 2013
*SlTTS1* *SlTTS2*L	12g006530 12g006520	Enzyme	Triterpenoid biosynthesis	*Sh*L	LA3917	Natural	Fruit cuticle wax triterpenoids; amyrin content and composition	Yeats et al., 2012a
*SlCHS1* *SlCHS2*L	09g091510 05g053550	Enzyme	Flavonoid biosynthesis	*Sl*L	MT/MM/GD	VIGS	Cuticle composition and properties; flavonoid, polysaccharide, cutin content and esters linkage; epidermal patterning	España et al., 2014; Heredia et al., 2015
*SlSHN1*L	03g116610	ERF	Regulation of wax biosynthesis	*Sl*L	MM	OE	Leaf cuticular wax; plant drought resistance	Al-Abdallat et al., 2014
*SlSHN3*L	06g053240	ERF	Regulation of cutin and wax biosynthesis	*Sl*L	MT	OE	Fruit and leaf cutin and wax content; fruit epidermal patterning; pathogen susceptibility	Shi et al., 2013; Buxdorf et al., 2014
*SlMIXTA-like*L	02g088190	MYB	Regulation of cutin and wax biosynthesis	*Sl*L	MT	RNAi silencing	Fruit cuticle thickness and properties; cutin content; epidermal patterning	Lashbrooke et al., 2015
*WOOLY*L	02g080260	HD-Zip IV	Regulation of wax biosynthesis	*Sl*L	LA3186	Natural/*Wo*L	Trichome initiation; leaf and fruit wax content	Xiong et al., 2020
*SlMYB31*L	03g116100	MYB	Regulation of wax biosynthesis	*Sl*L	AC	RNAi silencing/OE	Fruit cuticle properties; wax content	Xiong et al., 2020
*STICKY PEEL* *HD-ZIP IV*L	01g091630	HD-Zip IV	Regulation of epiderm metabolism	*Sl*L	M82	EMS/*cd2*L	Fruit and leaf cuticle properties; cutin, wax, flavonoid, anthocyanin content and/or composition; glandular trichomes; pathogen susceptibility	Isaacson et al., 2009; Martin et al., 2016
				*Sl*L	LA0759	Natural/*pe*L		Kimbara et al., 2012, 2013
				*Sl*L	LA2467			Nadakuduti et al., 2012
*YELLOW* *PINK FRUIT* *SlMYB12*L	01g079620	MYB	Regulation of flavonoid metabolism	*Sl*L	LA3189	Natural/*y*L	Fruit cuticle thickness and properties flavonoid accumulation in the cuticle; epidermal patterning	Adato et al., 2009
				*Sc*L	LA1480	Natural/*y*L		Ballester et al., 2010
				*Sl*L	MT	EMS/*pf*L		
*LYCOPENE CYCLASE b*L		Enzyme	Carotenoid metabolism	*Sl*L	MM	OE Arabidopsis AT3G10230	Extended shelf-life; fruit cuticle thickness and properties; ABA content; cutin and triterpenoid content	Diretto et al., 2020
*SlPP2C3*L	06g076400	Enzyme	Regulation of ABA metabolism	*Sl*L	MT	RNAi silencing/OE	Fruit cuticle properties; epidermal patterning	Liang et al., 2021
*GA2-OXIDASE*L	10g007570	Enzyme	GA catabolism	*Sl*L	LA1310	Natural/*fis1*L	Fruit firmness; cuticle thickness; cutin and wax content	Li et al., 2020
*TAGL1*L	07g055920	MADS box	Regulation of fruit ripening	*Sl*L	MM	RNAi silencing/OE	Fruit cuticle thickness and properties; cutin, wax, cell wall and phenolics composition and content; epidermal patterning	Giménez et al., 2015
*RIN*L	05g012020	MADS box	Regulation of fruit ripening	*Sl*L	AC	Natural/*rin*L	Long shelf-life; fruit cuticle composition and properties; cutin and wax composition	Kosma et al., 2010
*NAC-NOR ALCOBACA*L	10g006880	NAC	Regulation of fruit ripening	*Sl*L	DFD	Natural/*nor/alc*L	Long shelf-life; cuticle composition and properties; cutin and wax content and composition	Saladié et al., 2007; Romero and Rose, 2019
				*Sl*L	AC			Kosma et al., 2010
				*Sl*L	de Penjar			Kumar et al., 2018
*FUL1* *FUL2*L	06g06943003g114830	MADS box	Regulation of fruit ripening	*Sl*L	MT	RNAi silencing	Cuticle properties	Bemer et al., 2012
*CWP1* *FNC*L	04g082540		Unknown	*Sh*L	LA1777	Natural/*cwp*L	Cuticle fissures; fruit dehydration	Hovav et al., 2007; Chechanovsky et al., 2019
				*Sp*L	LA716	Natural		Zhang et al., 2021
*SlEZ2*L	03g044380	Enzyme	Histone methylation	*Sl*L	Wva106	RNAi silencing	Fruit brightness; cutin and wax content and composition	Boureau et al., 2016

^*^The table is focused on published studies in which the tomato cuticle was explicitly analyzed, as revealed by an extensive literature review.

a Locus and/or gene names are indicated when available in the cited references. *CYP*, *CYTOCHROME P450*; *GPAT*, *GLYCEROL-3-PHOSPHATE ACYLTRANSFERASE*; *ABCG*, *ATP-BINDING CASSETTE (ABC) G*; *CUS*, *CUTIN SYNTHASE*; *CER*, *ECERIFERUM*; *KCS*, *β-KETOACYL-COA-SYNTHASE*; *DCR*, *DEFECTIVE IN CUTICULAR RIDGES*; *TTS*, *TRITERPENE SYNTHASE*; *CHS*, *CHALCONE SYNTHASE*; *SHN*, *SHINE*; *HD-Zip IV*, *HOMEODOMAIN-LEUCINE ZIPPER IV*; *PP2C*, *PROTEIN PHOSPHATASE 2C*; *TAGL1*, *TOMATO AGAMOUS-LIKE1*; *RIN*, *RIPENING INHIBITOR*; *NOR*, *NON-RIPENING*; *FUL*, *FRUITFULL*; *CWP*, *CUTICULAR WATER PERMEABILITY*; and *FNC*, *FRUIT NETTED CRACKING*.

b The SGN *Solanum lycopersicum* (*Solyc*) identifier is indicated.

c ERF, Ethylene Response Factor; MYB, HD-Zip, MADS box, and NAC are transcription factors.

d *Sl*, *Solanum lycopersicum*; *Slc*, *Solanum lycopersicum var. cerasiformae*; *Sc*, *Solanum chmielewskii*; *Sh*, *Solanum habrochaites*; and *Sp*, *Solanum pennellii*.

e For natural mutations, the original species and Tomato Genetics Resource Center (TGRC) accession number of the genotype carrying the allelic variant studied are indicated. When not available in the cited references, the background genotype in which the mutation has been introgressed is indicated. MT, Micro-Tom; MM, Moneymaker; AC, Ailsa Craig; and GD, Gardener’s Delight.

f EMS, Ethyl methane sulfonate; RNAi, RNA interference; and OE, overexpression. The various names of the induced or natural alleles are indicated.

Linking gene to phenotype can be done using approaches known as (i) reverse genetics in which the function of a target gene is mainly determined by analyzing the phenotype of mutant or deregulated lines and (ii) forward genetics, in which tomato genetic diversity is first screened for phenotypes-of-interest and the underlying genes and their function are then identified.

## Exploring the Function of Cuticle-Associated Genes Through Reverse Genetics Approaches in Tomato

An obvious source of information on wax- and cutin-associated genes to study in tomato is the model plant Arabidopsis (Bernard and Joubes, 2013; Fich et al., 2016). Examples of the pertinence of this strategy are the conserved functions in Arabidopsis and tomato of the SHINE transcription factors (Shi et al., 2013; Al-Abdallat et al., 2014) and the recent demonstration that the SlABCG42 transporter, the tomato ortholog of the Arabidopsis ABCG PEC1 transporter, is functional and can transport various cutin precursors (Elejalde-Palmett et al., 2021).

For poorly characterized or unknown function genes, the information on where and when they are expressed provides the first cues on their possible role in cuticle formation. Gene expression has been explored in-depth in tomato fruit skin, a tissue which consists of the cuticle, the epidermis, and few layers of collenchyma cells underneath (Bargel and Neinhuis, 2005). Of special interest is the fruit expansion phase, before the onset of ripening, when rapid cuticle deposition occurs (Mintz-Oron et al., 2008; Petit et al., 2014). During the cell expansion phase, ploidy and volume of epidermal and sub-epidermal cells do not vary, whereas ploidy and cell volume undergo a dramatic increase in mesocarp cells (Joubès et al., 1999; Renaudin et al., 2017). In earlier studies, epidermis-expressed genes were identified *via* tissue-specific Expressed Sequence Tags (EST), microarrays (Lemaire-Chamley et al., 2005; Mintz-Oron et al., 2008), and proteome (Yeats et al., 2010). Several genes highlighted in these studies play prominent roles in cuticle formation, among which the *CUTIN SYNTHASE* (*CUS1*) catalyzing cutin polymerization (Girard et al., 2012; Yeats et al., 2012b) and the *SlMIXTA-like* regulating cuticle formation and epidermal patterning (Lashbrooke et al., 2015). Semi-quantitative RNA-seq coupled with laser microdissection (LMD) next allowed exhaustive inventory of gene transcripts expressed in plant tissues, including outer and inner fruit epidermis (Matas et al., 2011). Another original transcriptome-based approach used a chimera between two tomato species displaying genotype-specific E1 cell layer to provide a reference catalog of epidermis-specific genes (Filippis et al., 2013). Quantitative RNA-seq coupled with LMD later allowed the establishment of a quantitative tomato fruit gene atlas of developing fruit (Shinozaki et al., 2018). The resulting Tomato Expression Database (TEA) available at SGN^2^ can be mined for clusters of genes co-expressed in specific developmental stages and cell types including inner and outer epidermis, which gives precious information on genes functionally linked to cuticle formation. Gene expression profiling can be further combined with various multi-omics technologies, typically metabolome (Fernandez-Moreno et al., 2016) and proteome (Szymanski et al., 2017), and with large scale sequencing of genetic diversity (Szymański et al., 2020) to assign putative functions to the genes and explore their role in cuticle formation. Novel functions revealed by biochemical approaches, e.g., the cutin:cutin-acid endo-transacylase (CCT) enzyme activity (Xin et al., 2021), may also ultimately lead to the isolation of the encoding gene.

Technologies used for the functional analysis of cuticle-associated genes (Table 1) include Virus-Induced Gene Silencing (VIGS; Ballester et al., 2010; España et al., 2014), stable gene overexpression (e.g., Shi et al., 2013) and silencing *via* RNA interference (RNAi; e.g., Girard et al., 2012; Lashbrooke et al., 2015) and artificial miRNA (Adato et al., 2009). More recently, the efficient CRISPR/Cas9 system, which allows genome editing of single or multiple target genes (Rothan et al., 2019), has been successfully used to validate the influence of a GA2-oxidase on cuticle formation and fruit firmness (Li et al., 2020). Recent development of genome editing technologies offers the possibility to specifically target cuticle genes in the fruit (Feder et al., 2020) when rapid cuticle deposition takes place (Mintz-Oron et al., 2008) by using the fruit- and expansion phase-specific promoter *proPPC2* (Fernandez et al., 2009; Guillet et al., 2012). This further opens the perspective to specifically edit a cuticle gene in fruit epidermis, for instance by employing the promoter from the wax biosynthesis *SlCER6* gene (Vogg et al., 2004) that drives reporter expression in both inner and outer fruit epidermis (Mintz-Oron et al., 2008).

## Discovering Cuticle-Associated Genes Through Forward Genetics Approaches in Tomato

Though the list of cuticle-associated genes can be considerably shortened by mining existing databases and literature, their systematic analysis in planta may remain problematic because of gene redundancy, pleiotropic effect, etc. This may restrict the focus to known gene families, thus impeding the discovery of original gene functions. An alternative approach is to screen natural or artificially induced genetic diversity for phenotypes-of-interest, e.g., fruit surface defects induced by cutin deficiency (Isaacson et al., 2009; Petit et al., 2014), and then identify causal genetic variation.

### Using Natural Genetic Diversity for Linking Cuticle Phenotype to Gene

Wild tomato species are an important source of genes lost during domestication of tomato (Gao et al., 2019). The main tomato germplasm repository, from which seeds can be ordered, is the Tomato Genetics Resource Center (TGRC).^3^ It includes the phenotypic description of wild tomato relatives and of thousands of cultivated tomato accessions carrying spontaneous or artificially induced mutations. Natural diversity can be screened for cuticle structure and composition (Yeats et al., 2012a; Halinski et al., 2015), transpirational water loss (Fich et al., 2020), and for mutations, such as *positional sterile* (*ps*), which causes organ fusion by affecting the wax decarboxylation pathway (Leide et al., 2011) or *yellow* (*y*) resulting from a *SlMYB12* mutation (Adato et al., 2009; Ballester et al., 2010).

Most wild tomato relatives are easily crossed with tomato to generate segregating populations, e.g., the *Solanum pimpinellifolium*-derived population (Barraj Barraj et al., 2021), thus allowing the detection of cuticle Quantitative Trait Locus (QTL) and candidate genes. Genomic regions of wild species can be further fixed in an uniform *S. lycopersicum* genetic background to produce Introgression Lines (Ils) or Backcross Inbred Lines (BIls; Bazakos et al., 2017; Petit et al., 2017). The most extensively used ILs, derived from *Solanum pennellii* (Eshed and Zamir, 1995), enabled the rapid mapping of cuticle-associated QTLs and genes for leaf waxes (Ofner et al., 2016), wax alkane and amyrin content (Fernandez-Moreno et al., 2017), epidermal reticulation of green fruit (Cui et al., 2017), and fruit skin microfissuring (Zhang et al., 2021). Large scale transcriptome and metabolome analysis of fruit skin from 580 inbred lines further identified genes involved in flavonoid biosynthesis and fungal resistance (Szymański et al., 2020). *Solanum habrochaites*-derived lines allowed the identification of candidate genes for wax triterpenoids (Yeats et al., 2012a) and the isolation of the *CWP1* gene of unknown function responsible for skin microfissuring (Hovav et al., 2007; Chechanovsky et al., 2019). *Solanum chmielewskii* ILs were used to identify a *y* mutant (Ballester et al., 2010).

The effects on cuticle formation of natural genetic variants found in cultivated tomato were also explored. *STICKY PEEL* encodes a HD-Zip IV protein that regulates epidermis metabolism and additional cuticle-associated traits (Kimbara et al., 2012; Nadakuduti et al., 2012) while *WOOLY*, a different HD-Zip IV protein, regulates trichome initiation and wax biosynthesis (Xiong et al., 2020). *GA2-OXIDASE*, which was identified through a fruit firmness QTL analysis, is unexpectedly related to a cuticle thickness QTL (Li et al., 2020) and connects cuticle formation with gibberellin (GA) signaling. In addition, because of the strong impact of cuticle properties on tomato postharvest storage (Saladié et al., 2007), lines harboring the well-studied non-ripening mutations *rin* and *nor* (Wang et al., 2020) as well as several long shelf-life tomato varieties [“Delayed Fruit Deterioration” (DFD) cultivar., de Penjar types] carrying allelic variants of *NAC-NOR* (e.g., *alcobaca*) have been characterized with respect to cuticle composition and properties (Saladié et al., 2007; Kosma et al., 2010; Kumar et al., 2018; Romero and Rose, 2019). The complex interplay between fruit development and ripening, epidermal patterning and metabolism, and cuticle formation and properties, which was revealed by these studies, was further supported by independent studies of the ripening regulators *FUL1/FUL2* (Bemer et al., 2012) and *TAGL1* (Giménez et al., 2015).

### Using Artificially Induced Genetic Diversity for Linking Cuticle Phenotype to Gene

Several collections of artificially induced genetic diversity have been generated in cultivated tomato by transposon tagging (Meissner et al., 1997) or ethyl methanesulfonate (EMS) mutagenesis (Menda et al., 2004; Minoia et al., 2010; Saito et al., 2011; Just et al., 2013; Gupta et al., 2017). The two main mutagenized cultivars are the miniature Micro-Tom tomato (Meissner et al., 1997), which is well suited for laboratory use (Meissner et al., 1997; Just et al., 2013), and M82 (Menda et al., 2004), a processing tomato parent of the widely used *S. pennellii* ILs (Eshed and Zamir, 1995). Several *cutin-deficient* mutants were found in the M82 mutant collection (Isaacson et al., 2009) while more than 10 wax-altered and/or cutin-deficient *glossy* mutants were described in Micro-Tom (Petit et al., 2014). Many more mutants can be found by screening tomato mutant collections for obvious cuticle defects (Petit et al., 2014) and by browsing associated phenotypic databases (reviewed in Rothan et al., 2016), such as TOMATOMA^4^ (Saito et al., 2011). EMS mutant collections can also be screened by TILLING (Targeting Induced Local Lesions IN Genomes; Okabe et al., 2011) though mutated alleles of target genes are now efficiently generated by gene editing (Rothan et al., 2019).

As detailed in Table 1, mutant collections have been instrumental for: (i) discovering novel gene functions (*SlCUS1*; Yeats et al., 2012b); (ii) isolating allelic variants of genes implicated in wax biosynthesis (*SlCER6*; Vogg et al., 2004; Smirnova et al., 2013), cutin biosynthesis (*SlCYP86A69*; Shi et al., 2013; *SlGPAT6*; Petit et al., 2016) and polymerization (*SlCUS1*; Petit et al., 2014), and regulation of flavonoid biosynthesis (*SlMYB12*; Fernandez-Moreno et al., 2016); and (iii) deciphering the regulation of epidermis (*HD-Zip IV*; Isaacson et al., 2009).

## Concluding Remarks and Perspectives

Thanks to its thick astomatous fruit cuticle that is easy to study and the wealth of mutants and lines with altered expression of cuticle-related genes already available, tomato provides an excellent model for deciphering the molecular determinants of cuticle formation, structure, and properties. As detailed in Table 1, reverse and forward genetics approaches led to the isolation of tomato genes involved in several cuticle-associated biosynthetic pathways including wax biosynthesis (*SlCER6*, *SLTTS1*, and *SlTTS2*); cutin biosynthesis (*SlCYP86A69* and *SlGPAT6*), transport (*SlABCG36/42*), and polymerization (*SlCUS1* and *SlDCR*); and flavonoid biosynthesis (*SlCHS1* and *SlCHS2*). Besides genes encoding various transcription factors (SlSHN1 and SlSHN3, SlMIXTA-like, two HD-Zip IV, SlMYB31, and SlMYB12) or related to ABA (*LYCOPENE CYCLASE b* and *SlPP2C3*) and GA (GA2-OXIDASE) hormonal pathways were shown to regulate cuticle formation and its coordination with epidermal patterning.

Altogether these studies demonstrated the interest of both reverse and forward genetic approaches for discovering novel cuticle gene functions and for generating new plant material to study in-depth cuticle architecture, properties, and interaction with other cell wall components. To face new challenges in cuticle studies, several research avenues can be envisaged in the next future. Analysis of key cuticle genes should be extended to additional family members not yet analyzed for their role in cuticle deposition. An example is that of the GDSL-domain family to which belongs the cutin synthase CUS1 enzyme, whose function was discovered and confirmed through convergent studies in tomato (Table 1). The expression of different *CUS1* homologs is deregulated, sometimes oppositely, in several cutin-deficient mutants (Lashbrooke et al., 2015; Petit et al., 2016). By analogy with the recently demonstrated role of GDSL-domain proteins in suberin formation (Ursache et al., 2021), CUS1 homologs (Yeats et al., 2014) may fulfill opposite functions of cutin polymerization and degradation in fruit skin, thereby adapting cuticle formation to rapid fruit growth. Particular attention should also be given to enzymes from the poorly explored phenolic pathway in the epidermis which has evolutionary conserved roles in cuticle properties (Philippe et al., 2020b; Kriegshauser et al., 2021) and to proteins involved in the modification of cell wall polysaccharides (Philippe et al., 2020a). Cell wall enzymes and other proteins likely play central roles in cuticle structure and properties and in the coordination of cuticle deposition with organ development and epidermal patterning (Philippe et al., 2020b). So far, few studies have linked cuticle formation in tomato with hormones, except for ABA (Diretto et al., 2020; Liang et al., 2021) and GA (Li et al., 2020). The role of ABA is not surprising considering the importance of ABA signaling in the response to various stresses (Curvers et al., 2010; Liang et al., 2021), as established in tomato leaves (Martin et al., 2017). More unexpected is the role of GA, the effect of which on cuticle formation is far from being understood (Li et al., 2020). In view of its numerous roles in plant defense and development (Fenn and Giovannoni, 2021), ethylene is also a likely candidate for regulating cuticle formation in tomato fruit. The selection of new “guilt-by-association” target genes for these pathways and processes can be considerably aided by literature mining and/or exploitation of various genomic data (transcriptome, proteome, and metabolome) including co-expression analyses across various fruit cell types and developmental stages (Shinozaki et al., 2018) and across various plant species (Lashbrooke et al., 2016).

In addition to the approaches described above, which often requires previous knowledge of the gene, pathway or process to be targeted, the exploration of untapped genetic diversity, for example, tomato landraces (Conesa et al., 2020), is a way to get off the beaten tracks. This can be very rewarding but also lead to the isolation of challenging genes with no obvious or demonstrated link to the cuticle (Li et al., 2020), or even unknown function (Hovav et al., 2007). One bottleneck in such approach is the phenotyping of large collections (Petit et al., 2017). It can be easy for obvious cuticular defects (Hovav et al., 2007), more challenging for less evident changes (Petit et al., 2014), and complex when low or medium throughput technologies are required, e.g., for wax and cutin monomer analyses (Fernandez-Moreno et al., 2017). In the recent years, high resolution genotyping, whole genome sequencing-based strategies, such as mapping-by-sequencing (MBS) or QTL-seq (Garcia et al., 2016; Bazakos et al., 2017), accelerated gene isolation in tomato (Rothan et al., 2019). However, though identifying the causal mutation can be relatively straightforward in EMS mutants (Garcia et al., 2016), isolating causal genetic variations from natural diversity can be complex since it usually requires crossing the genotype-of-interest with a distant genotype. The subsequent co-segregation in the progeny of numerous cuticle traits unrelated to the trait-of-interest may make phenotyping very difficult and even prohibit high resolution mapping of the genetic variant (Rothan et al., 2016). Association mapping has emerged in the last decade as a powerful tool to discover linkages between gene polymorphism and variations in fruit traits by exploring natural genetic diversity. In tomato, sequencing of hundreds of accessions combined with fruit phenotyping pinpointed genetic variations associated with changes in fruit size, flavor, and color among which *SlMYB12* polymorphisms responsible for pink fruit color (Zhu et al., 2018). More recently, pan-genome analysis of hundreds of wild and cultivated tomato accessions further uncovered numerous unknown genes and genetic variations underlying fruit traits (Gao et al., 2019; Alonge et al., 2020). Phenotyping selected panels of sequenced accessions for cuticle-associated traits should yield numerous genetic variations controlling cuticle and skin formation and properties, and help identifying new functions involved in these processes. In addition to the genetic control of cuticle formation, the epigenetic regulation of wax and cutin biosynthesis will be worth exploring in the near future, as a preliminary study has shown that altering histone methylation status has a profound effect on the composition of tomato fruit cuticle (Boureau et al., 2016; Table 1).

## Accession Numbers

Sequence data from this article can be found in the SGN database under accession numbers found in Table 1.

## Author Contributions

CR wrote the manuscript. CB and DM revised and edited the manuscript. All authors contributed to the article and approved the submitted version.

## Conflict of Interest

The authors declare that the research was conducted in the absence of any commercial or financial relationships that could be construed as a potential conflict of interest.

## Publisher’s Note

All claims expressed in this article are solely those of the authors and do not necessarily represent those of their affiliated organizations, or those of the publisher, the editors and the reviewers. Any product that may be evaluated in this article, or claim that may be made by its manufacturer, is not guaranteed or endorsed by the publisher.

## Footnotes

^1^ https://solgenomics.net/

^2^ https://tea.solgenomics.net/

^3^ https://tgrc.ucdavis.edu

^4^ https://toamatoma.nbrp.jp
